# The succession of gut microbiota in the concave‐eared torrent frog (*Odorrana tormota*) throughout developmental history

**DOI:** 10.1002/ece3.10094

**Published:** 2023-05-20

**Authors:** Qingkai Shi, Yue Li, Shuaitao Deng, Huijuan Zhang, Huiling Jiang, Liang Shen, Tao Pan, Pei Hong, Hailong Wu, Yilin Shu

**Affiliations:** ^1^ Collaborative Innovation Center of Recovery and Reconstruction of Degraded Ecosystem in Wanjiang Basin Co‐founded by Anhui Province and Ministry of Education School of Ecology and Environment Anhui Normal University Wuhu China; ^2^ Shanghai Wildlife and Protected Natural Areas Research Center Shanghai China

**Keywords:** developmental history, environmental factors, gut microbiota, host factors, *Odorrana tormota*

## Abstract

The gut microbiota of amphibians plays a crucial role in maintaining health and adapting to various developmental stages. The composition of gut microbial community is influenced by the phylogeny, habitat, diet, and developmental stage of the host. The present study analyzed the microbiota in the intestine of *O. tormota* at 11 developmental stages (from the tadpole at Gosner stage 24 to the 3‐year‐old adult) using high‐throughput 16S rRNA sequencing. Alpha diversity index analysis of the microbiota revealed that the index decreased from tadpole at Gosner stage 24 to adult frog stage, remained stable during the adult frog stages, but increased significantly at the early metamorphosis and hibernation preparation stages. The gut microbiota structure is similar in adult frogs but differs significantly in other developmental stages. Furthermore, the dominant phyla of gut microbiota in tadpoles were Proteobacteria, Firmicutes, Actinobacteria, and Bacteroidetes, whereas those in adult frogs were Proteobacteria, Firmicutes, Bacteroidetes, and Verrucomicrobia. Host and environmental factors jointly affected the gut microbial diversity and community composition of *O. tormota*, but developmental stage, feeding habit, and habitat type had a more significant influence. The microbial community in the gut varies with the developmental stage of the host and constantly adapts to the survival requirements of the host. These findings advance our understanding of the evolutionary mechanism of amphibian gut microbiota in maintaining health homeostasis and adaptation.

## Graphical abstract

The highest of Alpha diversity index and complexity of microbial symbiotic network structure were observed in Gosner stage 24. Alpha diversity index decreased from tadpole at Gosner 24 development to 2‐year‐old adult frog stage. Alpha diversity index increased significantly at the early metamorphosis and hibernation preparation stages. The development stage was the most significantly correlated with gut microbial diversity and community changes.

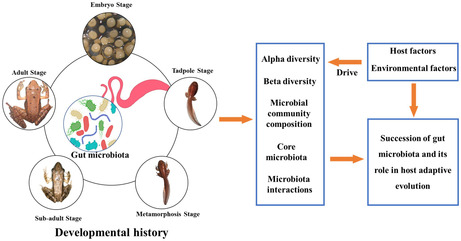

## 1 INTRODUCTION

Diverse populations of bacteria, archaea, fungi, protozoa, and viruses in the gastrointestinal tract are known as gut microbiota. These microorganisms are predominantly commensal or mutualistic. The gut microbiota participates in various physiological processes of the host, whereas the host provides a nutrient rich and suitable environment for the gut microbiota (Caruso et al., 2019). The gut microbiota has a significant effect on host health because of its crucial role in training the host's immunity, digesting food, regulating gut endocrine function and neurological signaling, modifying drug action and metabolism, eliminating toxins, and producing numerous compounds (Kruglov et al., 2013; Qin et al., 2010). Recently, research on gut microbiota has become a hot topic owing to its crucial role in health and disease.

As a keystone species linking aquatic and terrestrial environments, amphibians play a crucial role in the ecosystem. In a systematic study of the gut microbiota, amphibians received less attention than mammals and fish (Jiménez & Sommer, 2017). Changes in the gut microbiota are influenced by environmental factors (e.g., habitat type and temperature) and host factors (e.g., body weight and age) (Fontaine et al., 2018; Shu, Hong, Tang, et al., 2019; Shu, Hong, Yu, et al., 2019). Gut microbiota varies across individuals and developmental stages (Griffiths et al., 2018; Tong et al., 2019). Amphibians exhibit enormous variations in their life history (Morrison & Hero, 2003). For instance, tadpoles acquire the ability to breathe air and adapt to a terrestrial lifestyle (Kohl et al., 2013) through metamorphosis, involving apparent changes in a body structure (Brown & Cai, 2007; Dodd & Dodd, 1976). Meanwhile, the feeding habits of tadpoles have shifted from plankton and benthic organisms to insects (Chang et al., 2016). Hibernation is an adaptation to low‐temperature environments. By reshaping the gut microbiota and reducing the metabolic rate, the host can survive the winter at low temperatures and lacking food (Sonoyama et al., 2009). The unique life history of amphibians makes them an ideal system for examining differences in gut microbiota caused by changes in body structure, physiology, and immune system (Jiménez & Sommer, 2017). The gut microbial composition of certain tadpoles and frogs has been reported, and factors such as food, habitat type, and specific life stages (e.g., metamorphosis and hibernation) have a strong influence on gut microbiota (Tong, Cui, Hu, et al., 2020). Nevertheless, there are few systematic studies on the succession and adaptation strategies of amphibian gut microbiota throughout its developmental history.

*Odorrana tormota* (formerly *Amolops tormotus*) is distributed in a limited area of eastern China, located at the junction of the temperate zone, around mountain streams at elevations of 150–750 m. The species lays eggs in the streams, and the tadpoles live in the streams (Fei et al., 2009). Frogs are typically active at night and congregate on rocks in the stream or on trees, shrubs, and grass surrounding the stream. According to previous studies, tadpoles mainly feed on zooplankton and benthos, while adults primarily feed on insects (Fei, 1999; Li et al., 2008). Currently, the habitat environment, life history, food types, and genetic background of the concave‐eared frog have been investigated, making it an excellent model for studying the succession patterns of the intestinal microbiota of mountain stream frogs (Xiong et al., 2010; Shu et al., 2013; Shu et al., 2018). In this study, to clarify the succession process of the gut microbiota throughout the developmental history of *O. tormota*, we analyzed the composition, structure, and diversity of the gut microbiota at 11 different developmental stages based on 16S rRNA sequencing (from tadpole at Gosner stage 24 to the 3‐year‐old adult). Moreover, indicators of developmental stage, body weight, gut length, fasting status, feeding habits, habitat type, and environmental temperature were correlated with indicators of gut microbiota to investigate the key biotic and abiotic factors driving gut microbiota succession. This study provides a better understanding of the role of amphibian gut microbiota in maintaining health homeostasis and adaptation.

## 2 MATERIALS AND METHODS

### 2.1 Sample collection and measurement of host and environmental factors

All samples were collected from Jing County, Anhui Province, China (30.517500 N, 118.609444 E). Individuals were raised in the natural environment of the wild habitat from the fertilized egg stage to the end of the hibernation stage. *O. tormota* was fed according to the method of Shu et al. (2021). Adult frogs were captured randomly in mountain streams near our breeding ponds in mid‐June. The ages of adult frogs were determined by the bone age test (Matthews & Miaud, 2007). Tadpole samples were collected at Gosner stages 24 (T1), 31 (T2), 40 (T3), 42 (early metamorphosis, M1), and 45 (late metamorphosis, M2). Frog samples were collected at 20 days after metamorphosis (H0), pre‐hibernation stage (H1), hibernation stage (H2), end of hibernation stage (H3), 2‐year‐old adults (A1), and 3‐year‐old adults (A2). Three samples were randomly obtained from each developmental stage (Table S1). All samples were euthanized with MS‐222 overdose.

This study selected five host factors (developmental stage, gut length, body weight, fasting status, and feeding habits) and two environmental factors (habitat types and environmental temperature). During the dissection progress, gut length was measured using a Vernier caliper (JS168, Syntek, Deqing Shengtaixin Electronic Technology Co., Ltd., Huzhou, China), and body weight was measured using an electronic balance (JA1003B, Shanghai Yueping Scientific Instrument Co., Ltd., Shanghai, China). T1, T2, and T3 group samples were omnivorous tadpoles that mainly fed plankton and benthic organisms and lived in an aquatic environment. H0, H1, H2, H3, H3, A1, and A2 group samples were frogs that fed insects and lived mainly in terrestrial environments. M1, M2, and H2 group samples were in fasting status, whereas the other groups were in normal eating status. The ambient temperatures of the aquatic and terrestrial habitats were measured at 9 a.m. using thermometers placed 10 cm below the water surface and above the ground, including the hibernation stage. All data on host and environmental factors are presented in Table S2.

### 2.2 DNA extraction

Gut samples were collected in sterile tubes and immediately stored at −80°C until DNA extraction. DNA was extracted and analyzed for quality using the E.Z.N.A soil DNA kit (Omega Bio‐Tek, Norcross, GA, USA) and 2% agarose gel electrophoresis. All DNA samples were stored at −80°C until further analysis.

### 2.3 16S rDNA MiSeq sequencing and bioinformatics analyses

In 33 samples, the V3–V4 region of 16S rRNA gene was sequenced using the primer sequences 338F (ACTCCTACGG‐GAGGCAGCAG) and 806R (GGACTACHVGGGTWTCTAAT). The resulting PCR products were detected using 2% agarose gel electrophoresis and purified with the AxyPrep DNA Gel Extraction Kit (Axygen, New York, NY, USA). Purified PCR products were sequenced on the Illumina MiSeq platform (Illumina, San Diego, CA, USA), and specific amplicons were pooled in equimolar concentrations and subjected to paired‐end sequencing (2 × 300) on the Illumina MiSeq platform. Sequencing was performed by Majorbio Bio‐Pharm Technology Co. Ltd. (Shanghai Majorbio Bio‐Pharm Technology Co., Ltd., Shanghai, China) using standard protocols.

The paired‐end reads generated by MiSeq sequencing were spliced based on overlapping relationships and quality control. The QIIME2 software package selected representative sequences for each operational taxonomic unit (OTU). All representative sequences were compared and annotated with the Greengenes or Silva database, and OTUs annotated as chloroplast, mitochondria, and nonbacterial kingdoms were removed. OTUs were clustered at a 97% similarity cut‐off using Usearch (version 7.0 https://drive5.com/uparse/), and chimeric sequences were identified and removed using UCHIME. The RDP Classifier algorithm (https://rdp.cme.msu.edu/) was used to analyze the taxonomy of each 16S rRNA gene sequence against the Silva (SSU138) 16S rRNA database with a confidence threshold of 70%.

Sobs and Shannon indices were calculated using Mothur (v1.30.2) software for alpha diversity index analysis. Beta diversity was calculated using Bray–Curtis dissimilarity index between samples, computed using Qiime (v1.9.1). An analysis of nonmetric multidimensional scaling (NMDS) was performed using the “vegan” package in R to investigate the differences in microbial community composition between different developmental stages. The shared and unique OTUs among different groups were visualized using a Veen diagram. Network properties, including average path length, clustering coefficient, node degree, and betweenness centrality, were calculated using the “igraph” package in R and visualized using Cytoscape.

### 2.4 Statistical analysis

Posthoc LSD (Fisher's least significance difference) tests were used to determine the differences in alpha diversity between groups. The relationship between various factors and alpha diversity was explored using Spearman's rank correlation test. The multivariate effects of host and environmental factors on beta diversity were evaluated using PERMANOVA tests. Spearman's rank correlation test was performed to investigate the relationships between the microbial community, host, and environmental factors.

## 3 RESULTS

### 3.1 Summary of the sequencing data and alpha diversity of gut microbiota

A total of 46,053 high‐quality reads were obtained from 33 gut microbiota samples at 11 developmental stages. At a threshold of 97% sequence identity, 3904 operational OTUs from 49 phyla and 1120 genera were identified. The sample rarefaction curve (Figure S1) and the Shannon index rarefaction curve (Figure S2) indicated that the rarefied sequencing depths were sufficient to cover most bacterial communities in all samples.

**FIGURE 1:**
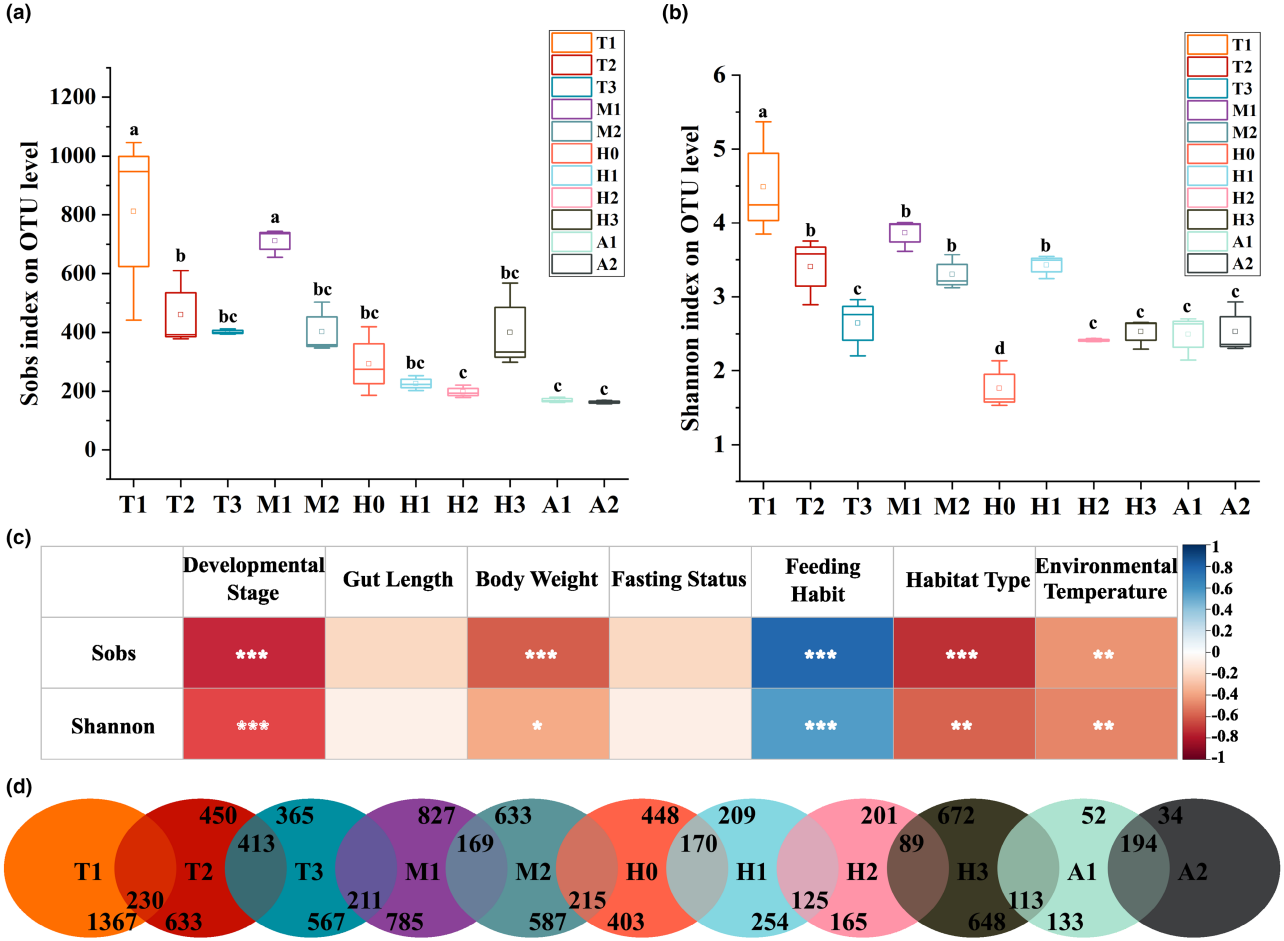
Comparison of sobs (a) and Shannon index (b) among different developmental periods of *O. tormota*. Bars in box and whisker plots represent median values. The different letters represent significant differences between groups (*p* < .05). Spearman correlations between host factors, environmental factors, and alpha diversity of gut microbiota in *O. tormota* (*.01 < *p* ≤ .05, **.001 < *p* ≤ .01, ***p ≤ .001) (c). The Venn diagram presented the number of unique and shared OTUs in the gut microbiota of *O. tormota* across developmental stages (d). T1, T2, T3, M1, and M2 refer stages for Gosner stages 24, 31, 40, 42 (early metamorphosis), and 45 (late metamorphosis), respectively. H0, H1, H2, H3, A1, and A2 refer stages for 20 days after metamorphosis, pre‐hibernation stage, hibernation stage, the end of hibernation stage, 2‐year‐old adults, and 3‐year‐old adults, respectively.

Sobs and Shannon indices were used to determine the richness and diversity of gut microbiota. According to the results, Sobs and Shannon indices were highest in the T1 group and decreased as development progressed (Figure 1a,b). There were significant differences in the Shannon index among T1, T2, and T3 groups, whereas there was a significant difference in the Sobs index between T1 and T2 groups. The Sobs and Shannon indices increased significantly in M1 group compared to those in T3 group. The Shannon index of H0 group revealed significant differences between M2 and H1 groups. The Shannon index was significantly higher in H1 group than in H2 group, whereas no significant differences existed between H2, H3, A1, and A2 groups.

Spearman's rank correlation test was conducted to investigate the associations between alpha diversity indices and various host and environmental factors (Figure 1c). The results demonstrated that Sobs index presented significant correlations with the developmental stage (*r* = −.804, *p* < .001), feeding habit (*r* = .761, *p* < .001), habitat type (*r* = −.761, *p* < .001), body weight (*r* = −.590, *p* < .001), and environmental temperature (*r* = −.448, *p* < .01). Furthermore, Shannon index revealed significant correlations with the developmental stage (*r* = −.603, *p* < .01), feeding habit (*r* = .665, *p* < .001), habitat type (*r* = −.665, *p* < .01), environmental temperature (*r* = −.487, *p* < .01), and body weight (*r* = −.363, *p* < .05).

### 3.2 Shared OTUs throughout development history

A Venn diagram was used to depict the overlap and unique OTUs across developmental stages (Figure 1d). A total of 1597, 863, 778, 996, 802, 618, 379, 290, 761, 246, and 228 OTUs were found in T1, T2, T3, M1, M2, H0, H1, H2, H3, A2, and A1 groups, respectively. Among these, 762, 189, 161, 430, 241, 187, 62, 42, 113, 8, and 9 OTUs were unique to each developmental stage. Nine OTUs were shared across all developmental stages (Table 1). Four core OTUs (OTU 3199, 5424, 357, and 2851) were present in at least 90% of the samples. Furthermore, comparing adjacent developmental stages revealed the highest proportion of overlapped OTUs (69.3%) between A1 and A2 groups, whereas the lowest proportion (9.3%) was observed between H2 and H3 groups.

**TABLE 1:** Share OTUs across developmental history.

OTU	Genus		Phylum
OTU1034	*Pseudomonas*		Proteobacteria
OTU3199	*Pseudomonas*		Proteobacteria
OTU5424	*Acinetobacter*		Proteobacteria
OTU869	*Kluyvera*		Proteobacteria
OTU357	*unclassified_f__Enterobacteriaceae*		Proteobacteria
OTU233	*Microbacterium*		Actinobacteria
OTU3351	*Microbacterium*		Actinobacteria
OTU2851	*g__[Eubacterium]_fissicatena_group*		Firmicutes
OTU5353	*Terrisporobacter*		Firmicutes

### 3.3 Interactions network analysis

**FIGURE 2:**
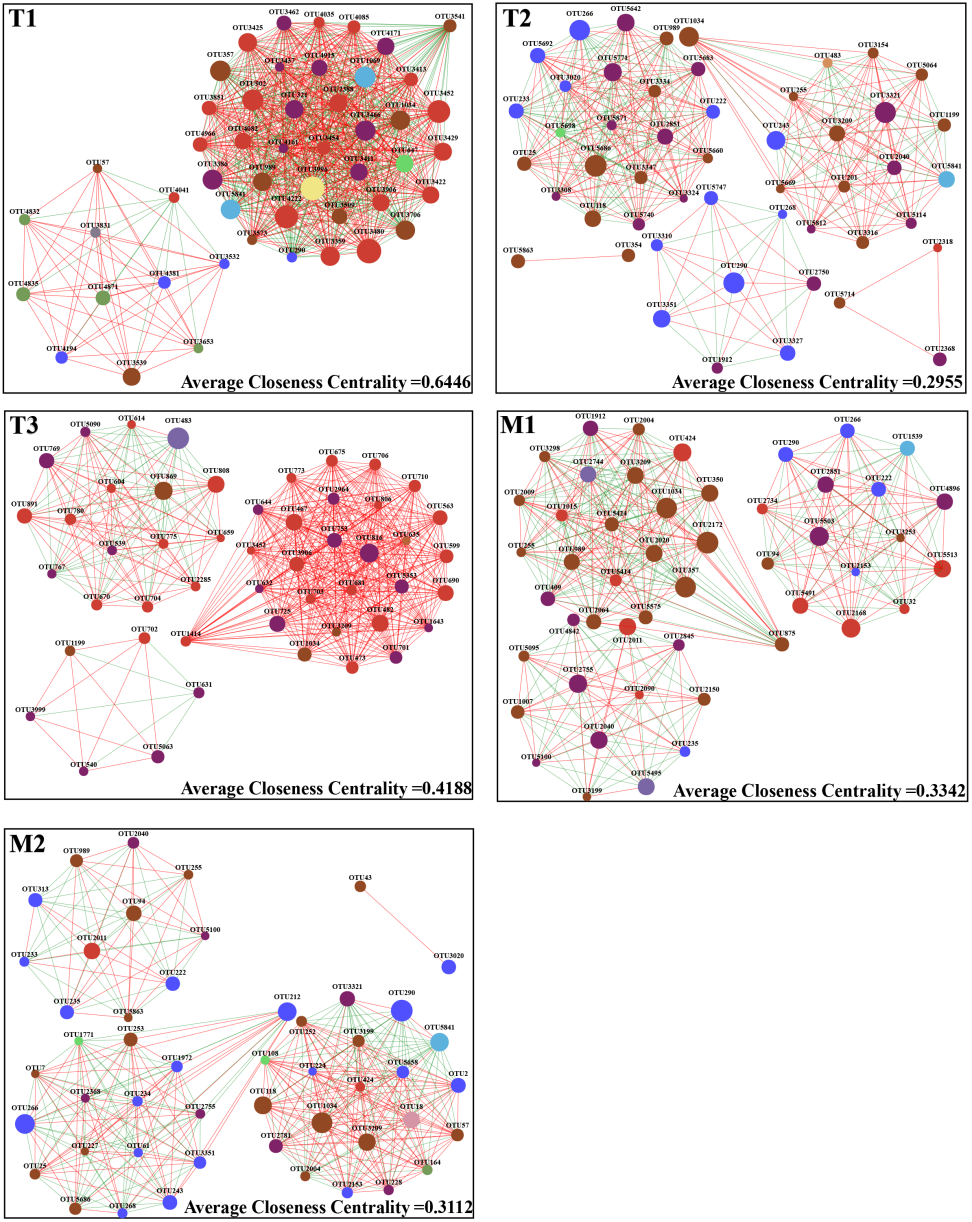
Network analysis of microbiota co‐interactions at tadpole stages. The nodes are colored by phylum membership and sized according to relative abundance. The red line represents a positive Spearman correlation, and the green line represents a negative correlation. Thickness of line represents strength of correlations.

The topological features of correlation networks were used to study the co‐interaction among microbiota. The results presented that the average closeness centrality in tadpole stages was 0.6446 (T1), 0.2955 (T2), 0.3112 (T3), 0.4118 (M1), and 0.3342 (M2) (Figure 2). The average closeness centrality in larvae frog stages was 0.3102 (H0), 0.3227 (H1), 0.3802 (H2), and 0.2891 (H3), and that in adult frog stages was 0.3367 (A1) and 0.3307 (A2) (Figures S3 and S4).

### 3.4 Beta diversity of gut microbiota

**FIGURE 3:**
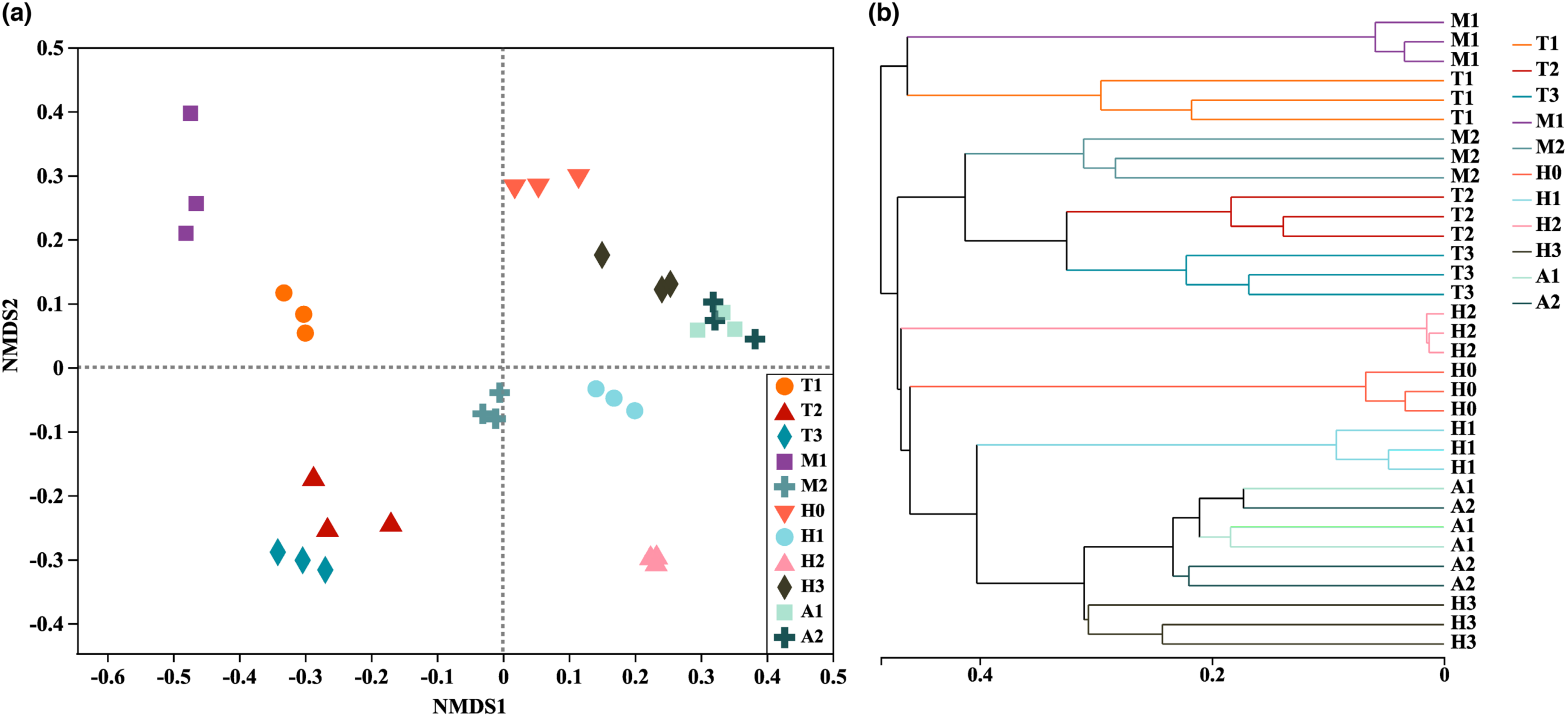
NMDS plot of microbial compositional structure based on Bray–Curtis dissimilarities (stress = 0.129, ANOSIM, *R* = .9752, *p* = .001) (a). Cluster analysis of microbial community structure from gut microbiota samples at different developmental stages (b).

The nonmetric multidimensional scaling (NMDS) ordination plot (based on Bray–Curtis distance matrix of OTU relative abundances) indicated significant differences in bacterial community structure for different groups (stress = 0.129, ANOSIM, *R* = .9752, *p* = .001; Figure 3a). The analysis revealed clear separations among the samples at various developmental stages, except for some overlap in A1 and A2 groups. Notably, the gut microbiota of H3 group was similar to those of A1 and A2 groups. Hierarchical clustering (Figure 3b) produced similar results, indicating that the gut microbiota of the samples became more similar after hibernation.

PERMANOVA at the OTU level was used to evaluate further the contribution of environmental and host factors to the variations of microbial compositional structure. The results demonstrated that the gut microbial community composition was significantly influenced by the developmental stage (*r* ^2^ = 19.4, *F* = 7.48, *p* < .05), feeding habit (*r* ^2^ = 17.7, *F* = 6.66, *p* < .05), habitat type (*r* ^2^ = 17.7, *F* = 6.66, *p* < .05), body weight (*r* ^2^ = 14.6, *F* = 4.66, *p* < .05), environmental temperature (*r* ^2^ = 13.1, *F* = 4.66, *p* < .05), gut length (*r* ^2^ = 11.6, *F* = 4.05, *p* < .05), and fasting status (*r* ^2^ = 6.6, *F* = 2.18, *p* < .05) (Table S3).

### 3.5 Community composition of gut microbiota at the phylum level

**FIGURE 4:**
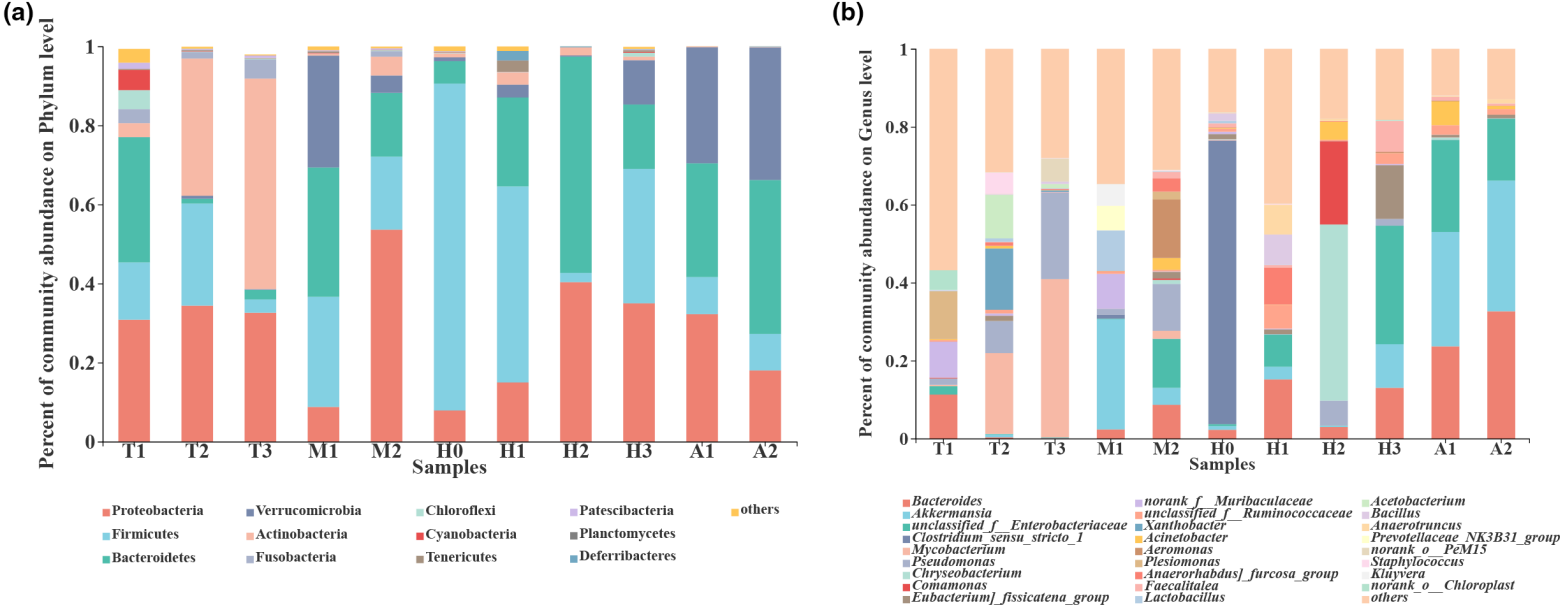
The relative abundance of phyla in the gut microbiota at different development stages (relative abundance >1%) (a). The relative abundance of genera in the gut microbiota at different development stages (relative abundance >5%) (b).

At the phylum level, 49 phyla were observed throughout developmental history from the gut microbiota of *O. tormota* (Table S4), whereas there were 12 phyla with a relative abundance of more than 1% (Figure 4a). Forty‐one phyla were observed in the T1 group. Bacteroidetes (31.69%), Proteobacteria (30.85%), Firmicutes (14.50%), Cyanobacteria (5.14%), Chloroflexi (4.81%), Fusobacteria (3.55%), Actinobacteria (3.52%), and Patescibacteria (1.55%) were main phyla in the T1 group, whereas the relative abundance of Cyanobacteria, Chloroflexi, and Patescibacteria was low (<1%) in other 10 developmental stages. In the T2 group, 25 phyla were observed. The relative abundance of Actinobacteria (3.52%–34.73%), Proteobacteria (30.85%–34.41%), and Firmicutes (14.50%–25.83%) was increased compared with T1 group, whereas that of Bacteroidetes (31.69%–1.22%) and Fusobacteria (3.55%–1.65%) was decreased. In T3 group, 25 phyla were observed. The relative abundances of Actinobacteria (34.73%–53.27%) and Fusobacteria (1.6%–4.85%) increased compared to those in T2 group, whereas those of Proteobacteria (34.41%–32.62%) and Firmicutes (25.83%–3.36%) were decreased (Figure 4a).

In M1 group, 21 phyla were observed. The relative abundance of Bacteroidetes (2.4%–32.71%), Verrucomicrobia (0.2%–28.30%), and Firmicutes (3.36%–27.87%) was increased compared with that in T3 group, whereas that of Actinobacteria (53.27%–0.52%) and Proteobacteria (32.62%–8.79%) was decreased. In M2 group, 25 phyla were observed. The relative abundances of Proteobacteria (8.79%–53.64%) and Actinobacteria (0.52%–4.77%) were higher than those in M1 group, whereas those of Bacteroidetes (32.71%–16.13%), Verrucomicrobia (28.30%–4.40%), and Firmicutes (27.87%–18.48%) were decreased. In H0 group, 24 phyla were observed. The relative abundance of Firmicutes (18.48%–82.70%) was increased compared with M2 group, whereas that of Bacteroidetes (16.13%–5.68%), Verrucomicrobia (4.40%–0.97%), Proteobacteria (53.64%–7.89%), and Actinobacteria (4.77%–0.99%) was decreased. Throughout the developmental history, the relative abundance of Firmicutes in H0 group reached the highest (82.70%), and that of Bacteroidetes reached the lowest (5.68%) (Figure 4a).

In H1 group, 24 phyla were observed. The relative abundance of Bacteroidetes (5.7%–22.50%), Proteobacteria (7.9%–14.98%), Verrucomicrobia (0.97%–3.26%), and Actinobacteria (0.99%–3.14%) was increased compared with the H0 group, whereas that of Firmicutes (82.70%–49.59%) was decreased. Furthermore, the relative abundance of Tenericutes (2.85%) and Deferribacteres (2.40%) was highest in the H1 group, whereas that of Tenericutes and Deferribacteres was lowest (<1%) in other developmental stages. In H2 group, 16 phyla were observed. The relative abundances of Proteobacteria (14.98%–40.36%) and Bacteroidetes (22.50%–54.73%) were higher than those in the H1 group, whereas those of Firmicutes (49.59%–2.33%) and Verrucomicrobia (3.26%–0.29%) were decreased. In H2 group, the relative abundance of Bacteroidetes reached the highest (54.73%), and that of Firmicutes reached the lowest (2.33%) throughout developmental history. Compared with H0 group, the abundance of these two phyla changed in opposite ways (Figure 4a).

In H3 group, 35 phyla were observed. The relative abundances of Firmicutes (2.33%–34.02%) and Verrucomicrobia (0.3%–11.20%) were higher than those in H2 group, whereas those of Bacteroidetes (54.73%–16.29%) and Proteobacteria (40.36%–35.00%) were decreased. In A1 group, 10 phyla were observed. The relative abundances of Bacteroidetes (16.29%–28.78%) and Verrucomicrobia (11.20%–29.36%) were higher than those in H3 group, whereas those of Proteobacteria (35.00%–32.26%) and Firmicutes (34.02%–9.38%) were decreased. In A2 group, eight phyla were observed. The relative abundances of Bacteroidetes (28.78%–38.94%) and Verrucomicrobia (29.36%–33.55%) were higher than those in A1 group, whereas those of Proteobacteria were decreased (32.26%–18.02%) (Figure 4a).

### 3.6 Community composition of gut microbiota at the genus level

At the genus level, 1120 genera were observed throughout the developmental history of the gut microbiota of *O. tormota* (Table S5), whereas 80 genera had a relative abundance of more than 1% (Figure 4b). In T1 group, 583 genera were observed. The relative abundance of *Plesiomonas* was highest (12.29%). In T2 group, 450 genera were observed. The relative abundance of *Mycobacterium* (0.34%–20.73%) was increased compared with T1 group, whereas that of *Plesiomonas* (12.29%–0.01%) was decreased. In T3 group, 409 genera were observed. The relative abundance of *Mycobacterium* (20.73%–40.62%) was higher than that in T2 group, whereas that of *Xanthobacter* was decreased (15.73%–0.33%) (Figure 4b).

In M1 group, 333 genera were observed. The relative abundance of *Akkermansia* (0.09%–28.30%) was higher than that in T3 group, whereas that of *Mycobacterium* (40.62%–0.00%) was decreased. In M2 group, 447 genera were observed. The relative abundance of *Aeromonas* (0.05%–15.01%) was higher than that in M1 group, whereas that of *Akkermansia* was decreased (28.30%–4.36%).

In H0 group, 338 genera were observed. The relative abundance of *Clostridium_sensu_stricto_1* (0.04%–72.79%) was higher than that in M2 group, whereas that of *Aeromonas* (15.01%–0.00%) was decreased (Figure 4b). In H1 group, 247 genera were observed. The relative abundance of *Bacteroides* (2.23%–15.17%) was higher than that in H0 group, whereas that of *Clostridium_sensu_stricto_1* (72.79%–0.04%) was decreased. In H2 group, 169 genera were observed. The relative abundance of *Chryseobacterium* (0.02%–45.21%) was higher than that in H1 group, whereas that of *Bacteroides* (15.17%–2.95%) was decreased. In H3 group, 337 genera were observed. The relative abundance of *unclassified_f__Enterobacteriaceae* (0.09%–30.40%) was higher than that in H2 group, whereas that of *Chryseobacterium* (45.21%–0.02%) was decreased.

In A1 group, 114 genera were observed. The relative abundance of *Akkermansia* (11.20%–29.36%) was higher than that in H3 group, whereas that of *[Eubacterium]_fissicatena_group* (13.76%–0.64%) was decreased. In A2 group, 106 genera were observed. The relative abundance of *Bacteroides* (23.65%–32.64%) was higher than that in A1 group, whereas that of *unclassified_f__Enterobacteriaceae* (23.64%–15.89%) was decreased (Figure 4b).

**FIGURE 5:**
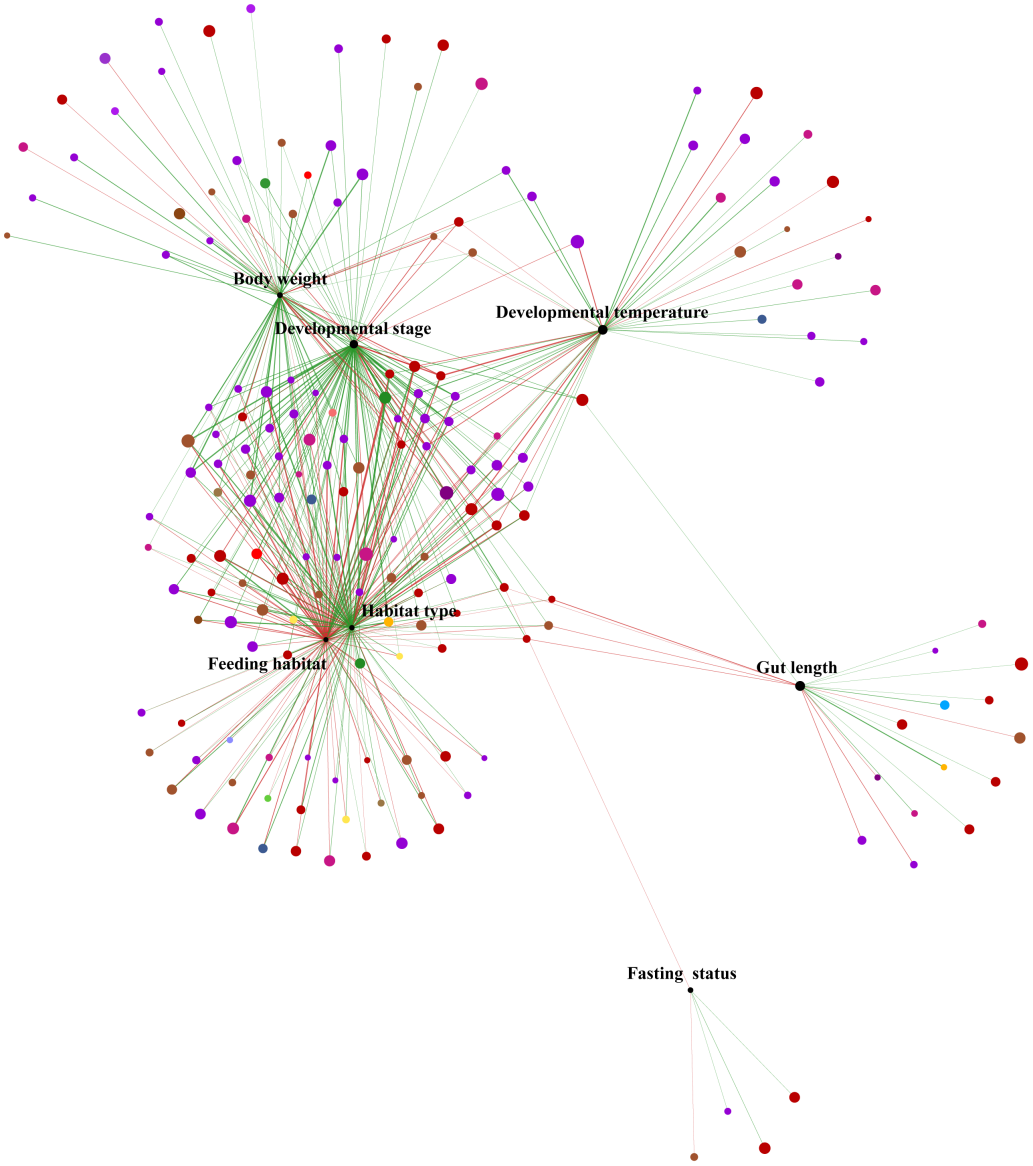
Network analysis of interactions (with Spearman index *r* > .5, *p* < .05) among microbiota community, host, and environmental factors. The red line represents a positive Spearman correlation, and the green line represents a negative correlation. Thickness of line represents strength of correlations.

In summary, there were differences in microbial community composition at the phylum and genus levels across different developmental stages. Greater differences in community composition at the phylum level were observed between T1 and T2, M1 and M2, and H1 and H2. Additionally, network analysis was used to determine the correlation between the relative abundances of various bacterial genera, host factors, and environmental factors. The results demonstrated that 109 genera were related to feeding habits and habitat type. Moreover, 103, 71, 45, 19, and 5 genera were related to developmental stage, body weight, environmental temperature, gut length, and fasting status, respectively (Figure 5).

## 4 DISCUSSION

The colonization and succession of gut microbiota and the driving factors in amphibians have rarely been systematically elucidated. In this study, the colonization, succession, and key drivers of the gut microbiota of *O. tormota* were resolved based on 16S rRNA sequencing and the determination of biotic and abiotic factors. The alpha diversity index decreased from tadpoles at Gosner stage 24 to adults but remained stable in adults. This trend was mainly driven by developmental stage, feeding habitat, and habitat type. The gut microbiota structure exhibits developmental adaptation and is relatively stable during adulthood. The microbial community in the gut varies with the developmental stage of the host and constantly adapts to the survival requirements of the host. This study contributed to our understanding of the role of the amphibian gut microbiota in maintaining health homeostasis and in the evolution of host adaptations.

Alpha diversity was used to evaluate the resilience, resistance, and stability of gut microbiota (Kim & Isaacson, 2015; Menni et al., 2017; Tong, Cui, Du, et al., 2020). This study found the highest Sobs and Shannon indices at Gosner stage 24. Except for the significant increase in the diversity index during the early metamorphosis and pre‐hibernation stages, alpha diversity was decreased from Gosner stage 24 to the 2‐year‐old frogs. Furthermore, alpha diversity in 2‐ and 3‐year‐old frogs tended to be relatively stable. The highest levels of alpha diversity were observed in tadpoles that had recently transitioned from their embryonic stage (Gosner stage 24), similar to the results of *Bufo gargarizans* and *Xenopus tropicalis* (Chai et al., 2018; Scalvenzi et al., 2021). This phenomenon may have occurred because the host immune system did not fully develop, preventing the formation of solid selection for colonization by foreign microorganisms (Giatsis et al., 2015; Yan et al., 2016). Furthermore, the highest complexity of microbial symbiotic network structure was observed at Gosner stage 24, suggesting that sufficient alpha diversity and complex interactions of the gut microbiota at Gosner stage 24 may lay the foundation for developing the tadpole gut microbiota (Coyte et al., 2015; Lawley & Walker, 2013). The increase in alpha diversity during early metamorphosis is similar to the results of *Xenopus tropicalis* (Scalvenzi et al., 2021), which may be related to some environmental microorganisms brought by consuming large quantities of food before metamorphosis in preparation for the fasting state. This study provides a foundation for selecting gut microorganisms during metamorphosis because of the high alpha diversity and complex interaction relationship of gut microorganisms at the early metamorphosis stage. Compared with frogs 20 days after metamorphosis, the diversity increased during the pre‐hibernation stage, which may be related to the fact that tadpoles consumed large quantities of food before hibernation to adapt to a fasting state and thus may have brought a large number of environmental microbiota via food (Kovács et al., 2007; Zhang et al., 2021). Although the alpha diversity of gut microbiota decreased during hibernation, it maintained a relatively complex co‐interaction network, resulting from the need to adapt to the balance between host physiology and gut microecology during the 4‐month hibernation stage in *O. tormota*. However, specific adaptive relationships require further research.

To gain insight into the role of microbiota in host health homeostasis and evolution, we investigated the drivers of alpha diversity formation in *O. tormota*. The present study found that host factors (developmental stage, feeding habit, and body weight) and environmental factors (habitat type and environmental temperature) were all associated with alpha diversity of gut microbiota, while developmental stage, feeding habit, and habitat type demonstrated a higher correlation with alpha diversity of gut microbiota. Previous studies have depicted that the gut microbiota alpha diversity of omnivorous animals is generally higher than that of specialized feeders (Wang et al., 2022). *O. tormota* tadpoles are omnivorous, whereas adult frogs are specialized feeders that mainly consume insects, such as Lepidoptera, Arachnida, Hymenoptera, and Orthoptera species (Fei, 1999; Shu, Hong, Tang, et al., 2019). It is generally observed that the alpha diversity of gut microbiota in animals varies with changes in their habitat (Huang et al., 2018). The alpha diversity of the gut microbiota of ornamented pygmy frogs (*Microhyla fissipes*) decreased after the transition from aquatic to terrestrial habitats (Zhang et al., 2020). In this study, a similar correlation was observed between the alpha diversity of gut microbiota, feeding habits, and habitat types in *O. tormota*. This further explains the reason for the low alpha diversity of gut microbiota in *O. tormota* during the frog stage, which is characterized by exclusive feeding habits and terrestrial habitat types. Moreover, the alpha diversity of gut microbiota usually changes with developmental stages (Yan et al., 2016). Developmental factors are a combination of physiology and body size differences at different developmental stages, ecological niche and habitat environments (Kohl & Yahn, 2016; Weng et al., 2016; Wiebler et al., 2018). These differences may influence the alpha diversity of gut microbiota. Therefore, the alpha diversity of gut microbiota in *O. tormota* showed the greatest correlation with developmental stage. Overall, host and environmental factors are drivers of the variation in alpha diversity of gut microbiota, where developmental stage, feeding habitat and habitat type have a greater impact on alpha diversity.

Beta diversity analysis can reveal the structural characteristics of the gut microbiota from *O. tormota* at various developmental stages (Anderson et al., 2011). In this study, the gut microbiota structure among adult frogs revealed the highest similarity, but the other developmental stages revealed significant differences. The structure of gut microbiota tends to be similar in the later stage of development, and the succession characteristics are similar to those of zebrafish (*Danio rerio*) and Atlantic cod (*Gadus morhua*) (Bakke et al., 2015; Stephens et al., 2016). This may be because post‐hibernation larvae frogs have a stable gut structure similar to that of adults and have similar ecological niches (Kohl et al., 2013). The drivers of beta and alpha diversity in the gut microbiota of *O. tormota* were similar, but the developmental stage, feeding habits, and habitat type may have a greater influence on beta diversity.

In this study, the dominant phyla of *O. tormota* tadpole were Proteobacteria, Firmicutes, Actinobacteria, and Bacteroidetes, which are incompletely consistent with *Lithobates clamitans*, *Lithobates catesbeianus*, and *Rana pipiens* tadpoles (Kohl & Yahn, 2016; Tong et al., 2019; Warne et al., 2017). The difference between different species may be attributed to habitat and host phylogeny (Shu, Hong, Tang, et al., 2019; Shu, Hong, Yu, et al., 2019). Furthermore, different tadpole species have different feeding habits, and the food composition of carnivorous species differs from that of herbivorous species (Levis et al., 2015). *O. tormota* tadpoles live in mountain streams, with few phytoplankton species and a relatively large number of benthos species (Kashinskaya et al., 2018; Schröder et al., 2013). According to literature research and anatomical observation, the gut contents of *O. tormota* tadpoles were full of Zooplankton and benthos (Li et al., 2008). The microorganisms carried by zooplankton and benthos species may be the primary source of the gut microbial composition of *O. tormota* tadpoles.

In this study, differences were found in the composition of the gut microbiota of *O. tormota* at all 11 developmental stages. Particularly, there were greater differences in the gut microbial composition of *O. tormota* between Gosner stage 24 and Gosner stage 30, Gosner stage 40, and Gosner stage 42, as well as before and during hibernation. Previous studies have demonstrated that Actinobacteria can form multienzyme complexes that aid the digestion of ingested carbohydrates by the host and participate in the synthesis of antibiotics (Qiao et al., 2019; Shu, Hong, Tang, et al., 2019). Therefore, compared to Gosner stage 24, the increased relative abundance of Actinobacteria at Gosner stage 31 may facilitate gut digestion and suppress pathogenic bacteria. Previous studies have demonstrated that *Akkermansia* can convert intestinal mucin into short‐chain fatty acids without intestinal nutrients (Weng et al., 2016). *Akkermansia* could also provide energy and affect intestinal immunity and barrier function (Plöger et al., 2012; Rooks & Garrett, 2016). Therefore, compared with the stage of early metamorphosis, the relative abundance of *Akkermansia* in the gut of *O. tormota* increased sharply at Gosner stage 42, which may provide energy substances and improve the immune for *O. tormota* in the fasting status during the stage of metamorphosis so that *O. tormota* can survive healthily at the special developmental stage of metamorphosis. Compared with the stages before metamorphosis, the relative abundance of some chitinase‐producing genera, such as *Bacillus*, *Parabacteroides*, and *Enterococcus*, were increased at the stages after metamorphosis completion. Increased relative abundance of chitinase‐producing genera may be attributable to the shift in the main food of *O. tormota* to chitin‐rich insects (Dhole et al., 2022; Shu, Hong, Tang, et al., 2019). Previous studies demonstrated that a high abundance of Firmicutes and Bacteroidetes and a high ratio of Firmicutes to Bacteroidetes increased the host's capacity to facilitate absorption or storage for the host (Murphy et al., 2010). In this study, the ratio of Firmicutes to Bacteroides in the gut of *O. tormota* was highest at 20 days after metamorphosis, the stage of preparation for hibernation, and the stage of end‐hibernation, but the ratio was the lowest at the hibernation stage. *O. tormota* needed to absorb or store large amounts of energy to cope with fasting or to recover quickly from fasting when it was at the stage of 20 days after metamorphosis, the stage of preparing for hibernation, and the stage of end‐hibernation. This low proportion may be related to the inability to absorb external nutrients at the hibernation stage. Furthermore, the relative abundance of *Pseudomonas* was higher at the hibernation stage. *Pseudomonas* can produce enzymes that hydrolyze urea, which may help *O. tormota* maintain the urea nitrogen cycle balance at the hibernation stage (Wiebler et al., 2018).

The composition of amphibian gut microbiota is usually influenced by multiple host and environmental factors (Tong, Cui, Hu, et al., 2020). In this study, we found that host factors, such as (developmental stage, feeding habits, fasting status, body weight, and gut length), as well as environmental factors (environmental temperature and habitat type) were all associated with gut microbial composition. Among these factors, feeding habits, habitat type, and developmental stage correlated more with gut microbial composition. *O. tormota* exhibits differences in feeding habits across different developmental stages. Tadpoles mainly feed on zooplankton and benthos, whereas adults primarily feed on insects. The feeding habits of *O. tormota* differ across different developmental stages. For example, tadpoles are omnivorous during the aquatic stage, while larvae and adult frogs primarily feed on insects (Shu, Hong, Tang, et al., 2019). As the developing body size gets larger, the type of food for *Odorrana schmackeri* also increases (Wu et al., 2015). As developing individuals adapt to a more complex ecological niche, the types of food acquired become more abundant (Huang et al., 2018). Different types of food can affect the gut microbiome by altering the nutritional environment in which gut microbes grow and by carrying different environmental microbes to colonize the gut (Derrien & van Hylckama Vlieg, 2015; Rinninella et al., 2019). Consequently, changes in the type of food acquired by *O. tormota* during development may affect the succession of gut microbial composition. *Polypedates megacephalus* altered its gut microbial composition after transitioning from aquatic to terrestrial habitats (Weng et al., 2016), and this alteration may be strongly associated with differences in the microbial composition of the habitat environment from which the gut microbiota originates (Cui et al., 2022). Differences in habitat environmental factors may also bring about other factors affecting gut microbial composition, which must be further explored. Additionally, gut microbial composition usually varies with developmental stage (Kohl & Yahn, 2016; Weng et al., 2016). During amphibian development, they undergo changes in physiology, body size, ecological niche, and habitat environment (Kohl & Yahn, 2016; Weng et al., 2016; Wiebler et al., 2018), all of which may affect the composition of gut microbiota. In summary, host and environmental factors affected the composition of the gut microbiota of *O. tormota*, with the effects of diet, habitat type, and developmental stage being more significant.

Although gut microbial composition changed significantly during development, 3904 OTUs, nine shared OTUs, and four core OTUs were found throughout the entire developmental history. A total of 49 shared OTUs and 10 core OTUs were found at all developmental stages of *Rana dybowskii*, speculating that there may be a strong association with the host (Tong et al., 2019). Furthermore, the four core OTUs have demonstrated high stability during the transition from freshwater to saltwater in Atlantic salmon (*Salmo salar*) (Rudi et al., 2018). The presence of core gut microbiota between reared sea bream (*Sparus aurata*) and sea brass (*Dicentrarchus labrax*) was independent of diet and geographic location (Hu et al., 2018; Nikouli et al., 2018). The core bacteria of forest musk deer (*Moschus berezovskii*) remained relatively stable throughout the four seasons. It has been suggested that for frog gut microbiota, these communities reflect the host habitat and are influenced by host‐specific selective forces (Tong et al., 2019). Therefore, although major changes were observed in *O. tormota* gut microbiota, the stability of core OTUs throughout the frog's developmental history may indicate a strong host association in the frog's gut.

## 5 CONCLUSION

This study systematically analyzed the succession rules and driven factors of *O. tormota* gut microbiota and explored adaptive strategies of *O. tormota* to different developmental stages (such as metamorphosis and hibernation) from the perspective of gut microbiota. This study revealed that, except for the stage of early metamorphosis and the stage of preparing for hibernation, alpha diversity was decreased from tadpoles at Gosner stage 24 to adult frogs and remained stable in adult frogs. By contrast, except for the most similar gut microbiota structure between adult frogs, the gut microbiota community structure of larvae frogs which finished hibernation was relatively similar to that of adult frogs, whereas there were significant differences in other developmental stages. The analysis revealed that host factors and environmental factors jointly affected the gut microbial diversity of *O. tormota*, but the developmental stage, feeding habit, and habitat type exerted a greater influence. Microbial composition was constantly adapting to the survival requirements of developmental stages, but there were four stable core OTUs throughout developmental history that might be strongly related to the basic survival requirements of *O. tormota*. This study contributes to our understanding of the evolutionary mechanism of amphibian gut microbiota in maintaining health homeostasis and adaptation.

## AUTHOR CONTRIBUTIONS

**Qingkai Shi:** Data curation (equal); methodology (equal); writing – original draft (equal). **Yue Li:** Methodology (equal). **Shuaitao Deng:** Methodology (equal). **Huijuan Zhang:** Methodology (equal). **Huiling Jiang:** Methodology (equal). **Liang Shen:** Software (equal); supervision (equal). **Tao Pan:** Software (equal); supervision (equal). **Pei Hong:** Formal analysis (equal); supervision (equal); writing – original draft (equal); writing – review and editing (equal). **Hailong Wu:** Conceptualization (equal); funding acquisition (equal); project administration (equal); supervision (equal); writing – review and editing (equal). **Yilin Shu:** Conceptualization (equal); data curation (equal); funding acquisition (equal); methodology (equal); project administration (equal); software (equal); supervision (equal); writing – original draft (equal); writing – review and editing (equal).

## Supporting information


Figure S1.

Figure S2.

Figure S3.

Figure S4.

Data S1.

Data S2.

Tables S1.–S5.

## Data Availability

The original 16S rRNA sequence data are available in the NCBI Sequence Read Archive under accession number PRJNA917058.
