# Supplementary figures and images for: The succession of gut microbiota in the concave‐eared torrent frog (Odorrana tormota) throughout developmental history
Source: Ecol Evol. 2023 May 20;13(5):e10094. doi: 10.1002/ece3.10094 (PMC10199338; doi:10.1002/ece3.10094)

Rarefaction curves

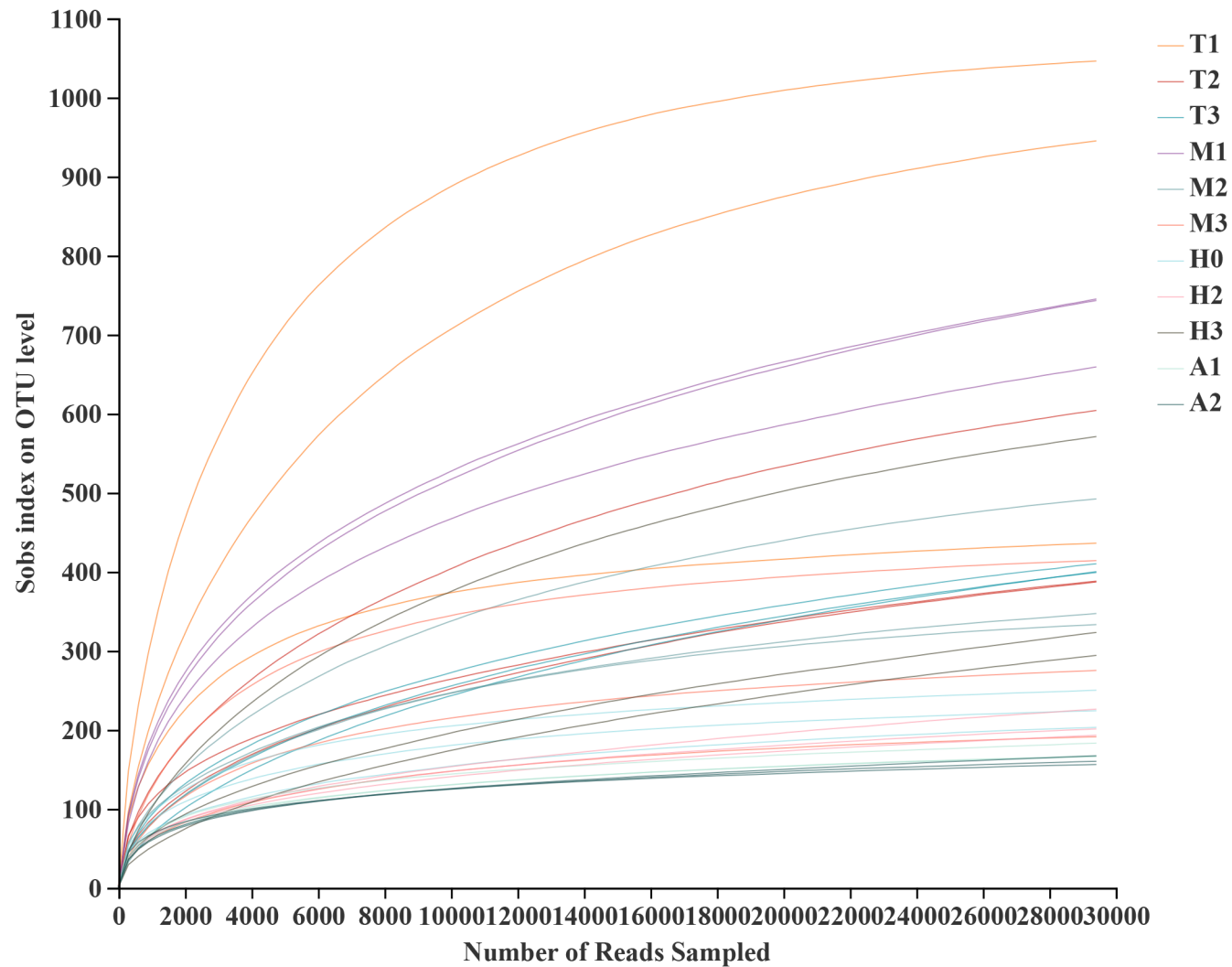

Supplement: Supplementary file 1 — Figure S1. [file ECE3-13-e10094-s001.pdf]

Shannon curves

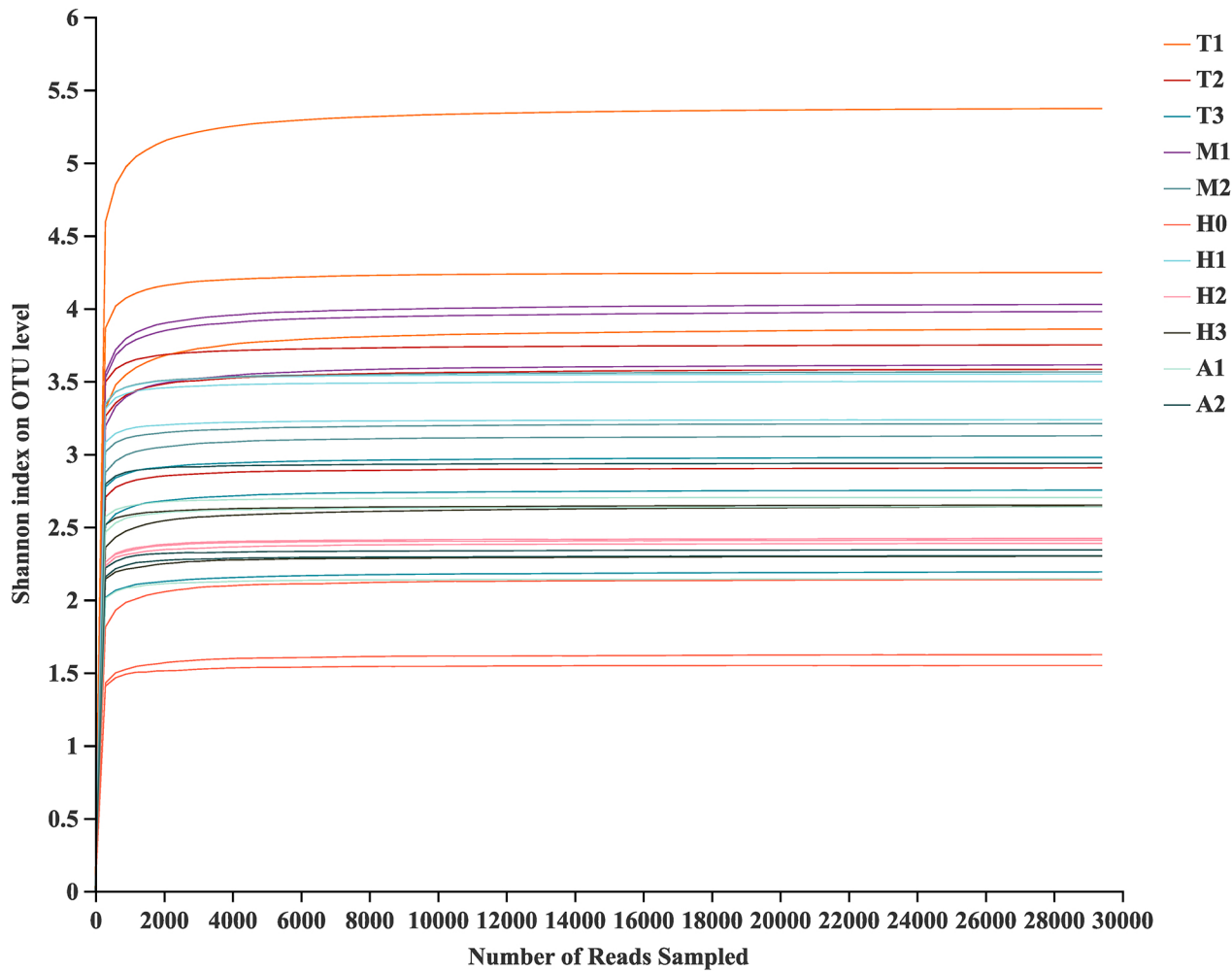

Supplement: Supplementary file 2 — Figure S2. [file ECE3-13-e10094-s005.pdf]

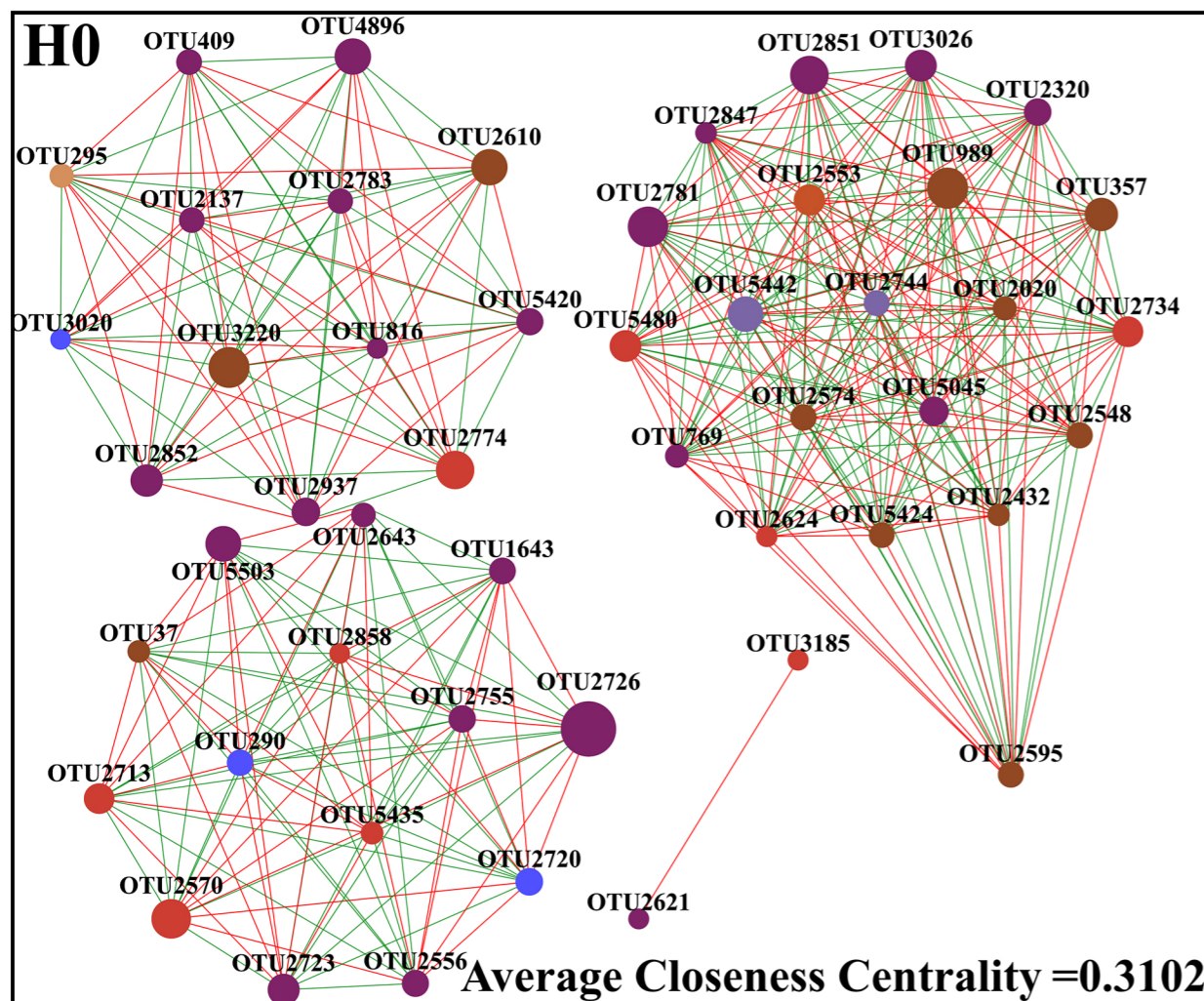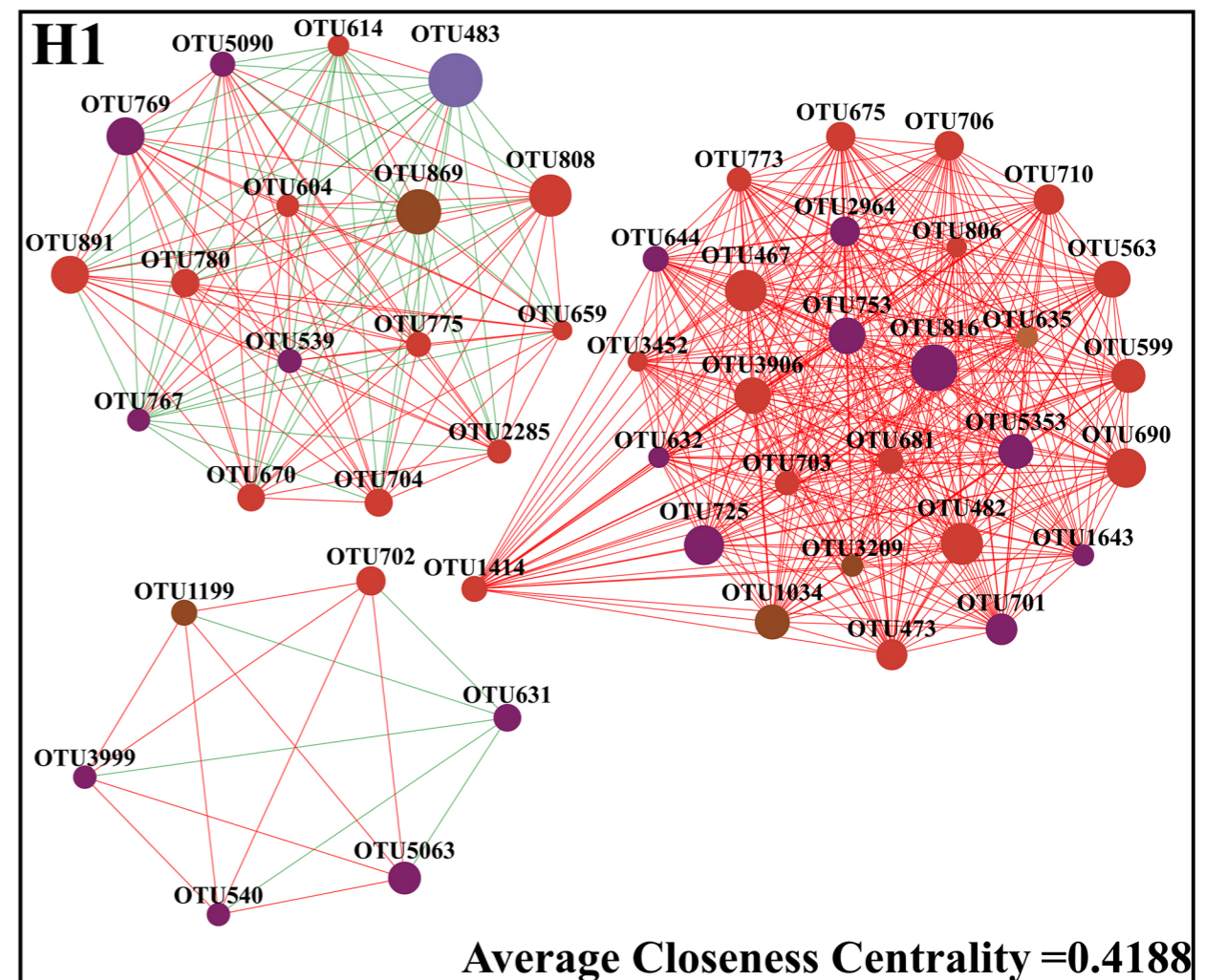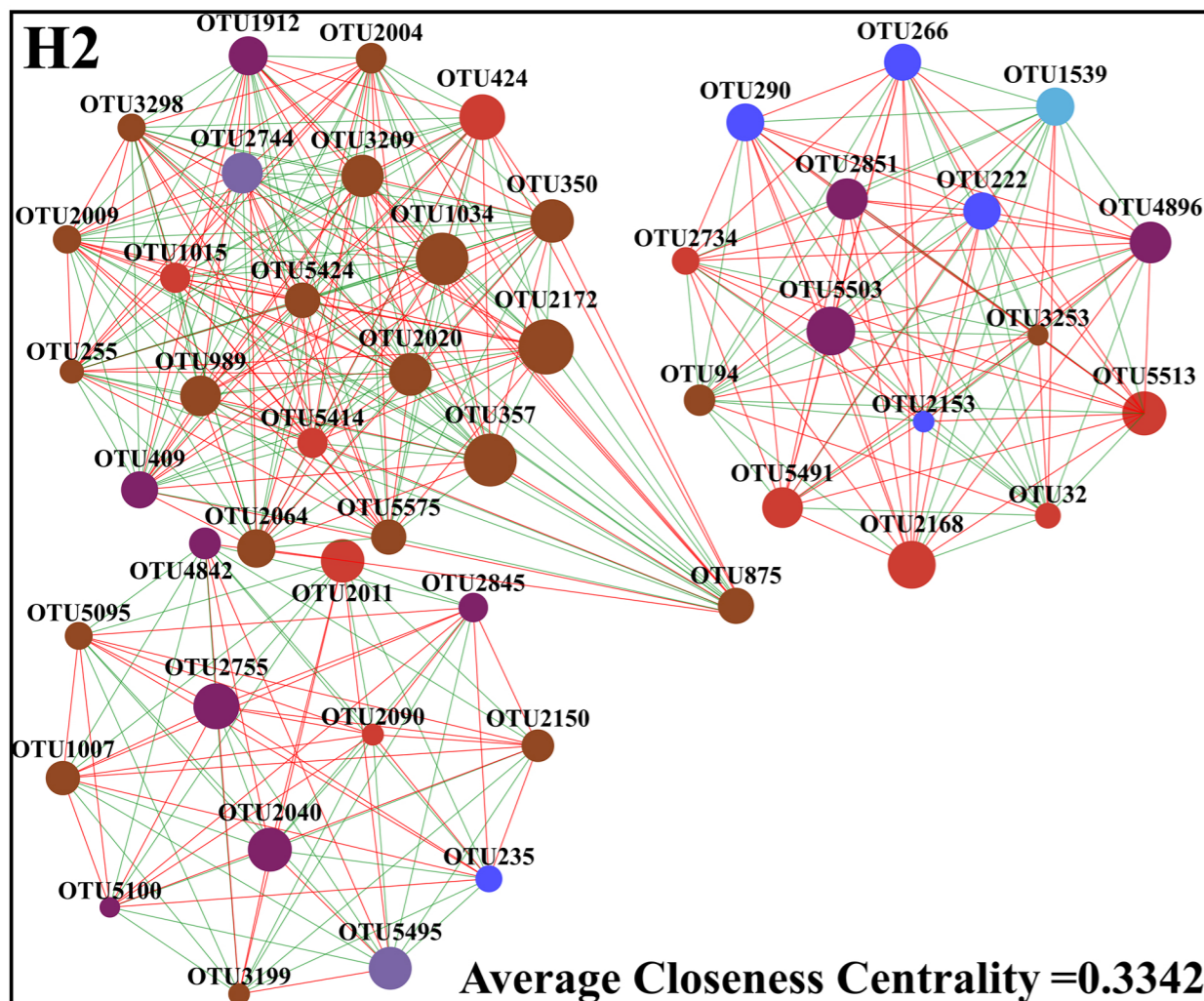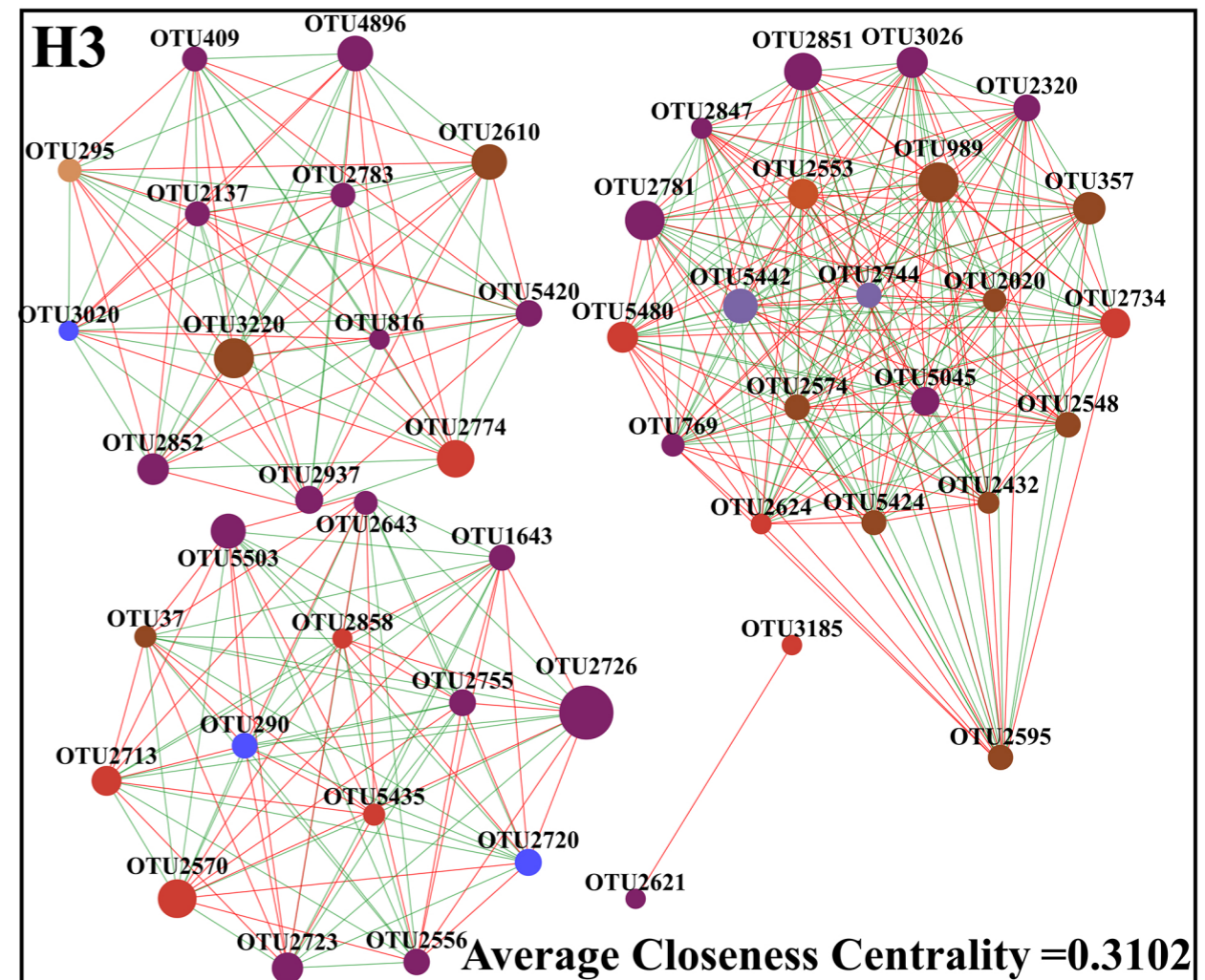

Supplement: Supplementary file 3 — Figure S3. [file ECE3-13-e10094-s002.pdf]

**A1**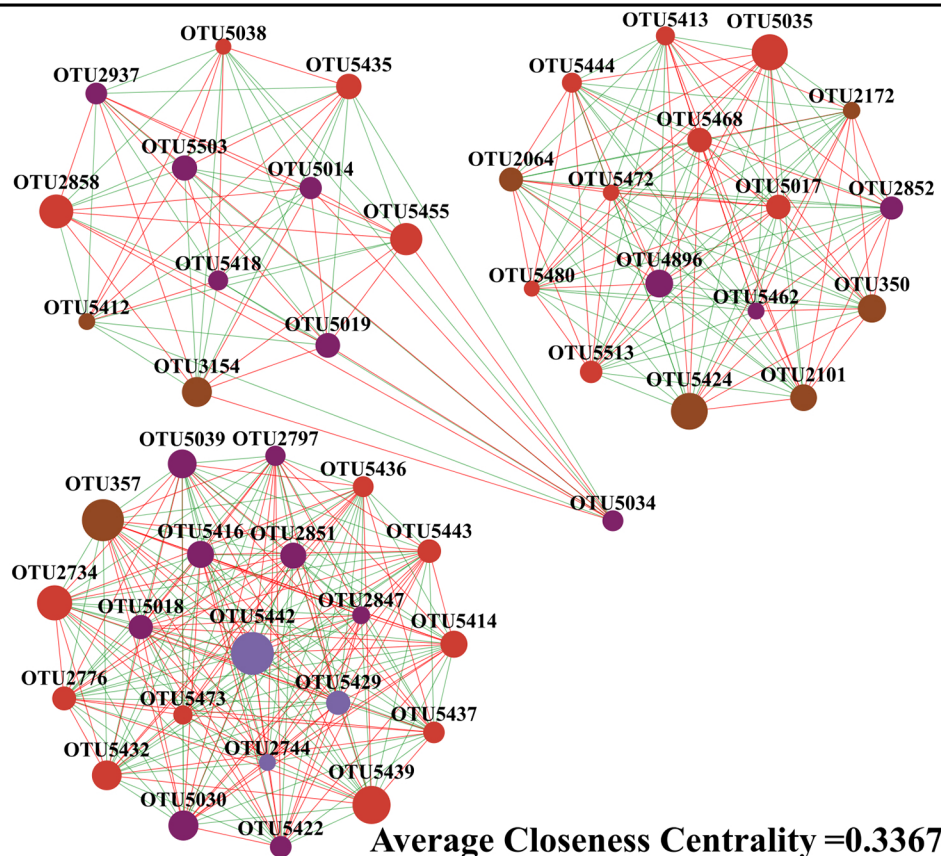**A2**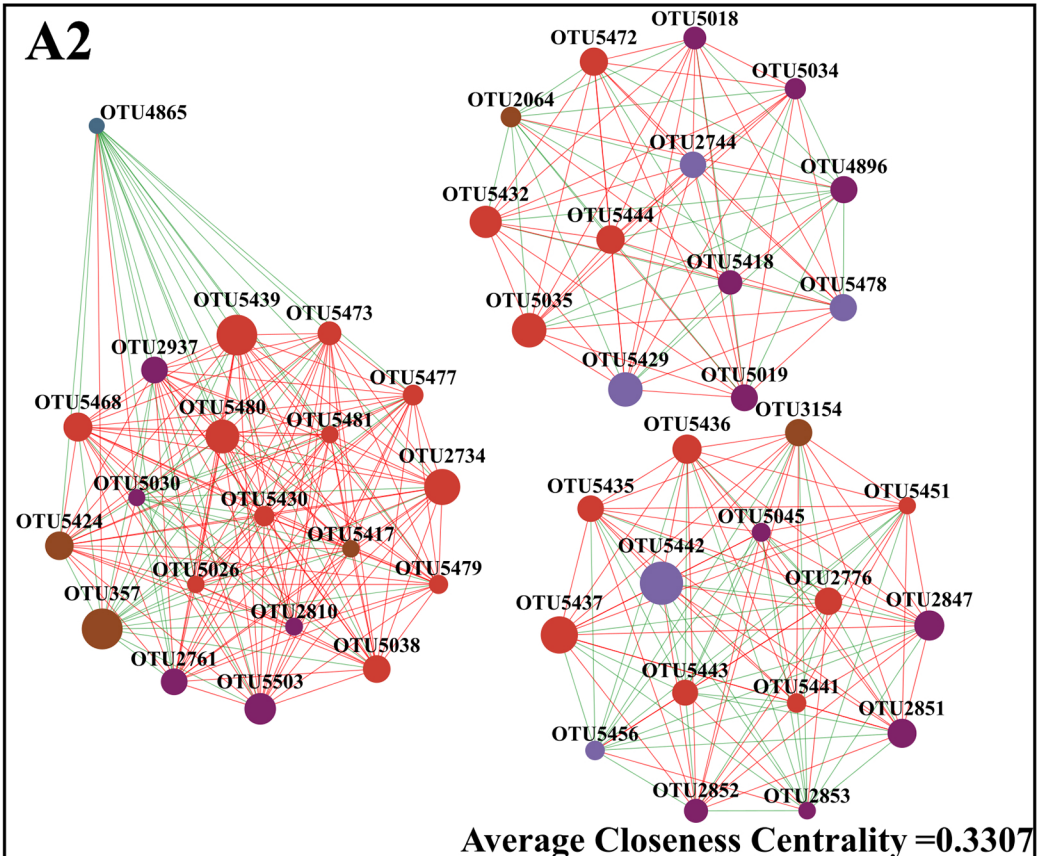

Supplement: Supplementary file 4 — Figure S4. [file ECE3-13-e10094-s003.pdf]
