# Supplementary material for: The succession of gut microbiota in the concave‐eared torrent frog (Odorrana tormota) throughout developmental history
Source: Ecol Evol. 2023 May 20;13(5):e10094. doi: 10.1002/ece3.10094 (PMC10199338; doi:10.1002/ece3.10094)
Supplement: Supplementary file 5 — Data S1. [file ECE3-13-e10094-s006.doc]

**Supplementary materials**

The succession of gut microbiota in the concave-eared torrent frog (*Odorrana tormota*) throughout developmental history

Qingkai Shi^1^, Yue Li^1^, Shuaitao Deng^2^, Huijuan Zhang^1^, Huiling Jiang^1^, Liang Shen^1^, Tao Pan^1^, Pei Hong^1^, Hailong Wu^1^*, Yilin Shu^1^*

*^1^ Collaborative Innovation Center of Recovery and Reconstruction of Degraded Ecosystem in Wanjiang Basin Co-founded by Anhui Province and Ministry of Education, School of Ecology and Environment, Anhui Normal University, Wuhu 241002, China*

*^2^ Shanghai Wildlife and Protected Natural Areas Research Center, Shanghai, 200336, China*

***** Corresponding authors:

| Prof. Hailong Wu  School of Ecology and Environment, Anhui Normal University, Wuhu, China  E-mail: whlong@mail.ahbu.edu.cn | Dr. Yilin Shu  School of Ecology and Environment, Anhui Normal University, Wuhu, China  E-mail: yilinshu@ahnu.edu.cn |
| --- | --- |

**Table and figure legends**

**SI Fig. S1** Sample rarefaction curve.

**SI Fig. S2** Shannon index rarefaction curve.
**SI Fig. S3** Network analysis of microbiota co-interactions at larval frog stages. The nodes are colored by phylum membership and sized according to relative abundance. The red line represents a positive Spearman correlation, and the green line represents a negative correlation. Thickness of line represent strength of correlations.
**SI Fig. S4** Network analysis of microbiota co-interactions at adult frog stages. The nodes are colored by phylum membership and sized according to relative abundance. The red line represents a positive Spearman correlation, and the green line represents a negative correlation. Thickness of line represent strength of correlations.

**Table S1** Sample grouping information.

**Table S2** Host and environmental factors information.

**Table S3** Relationship of host and environmental factors on beta diversity.

**Table S4** Relative abundance at the phylum level

**Table S5** Relative abundance at the genus level


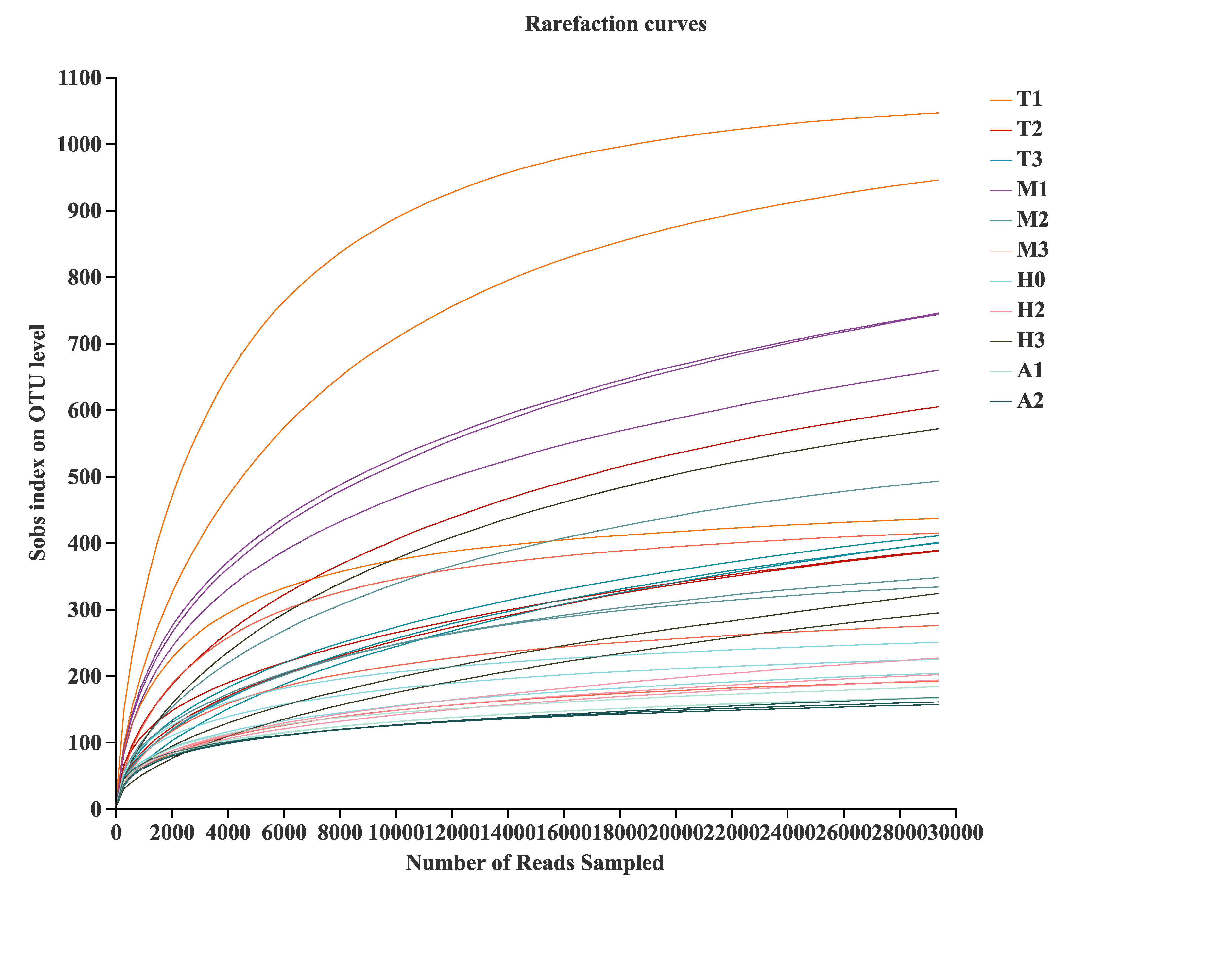


SI Fig. S1


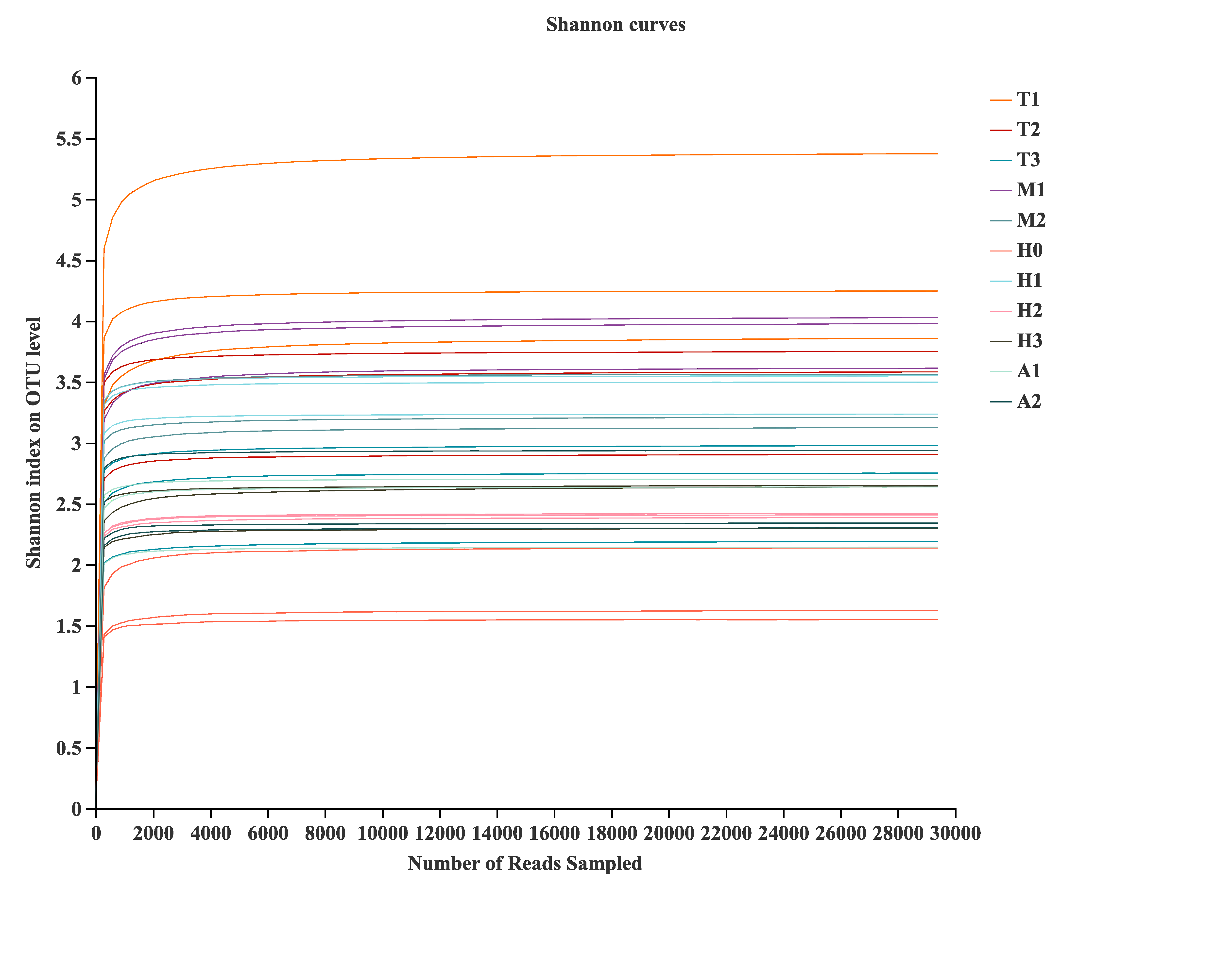


SI Fig. S2


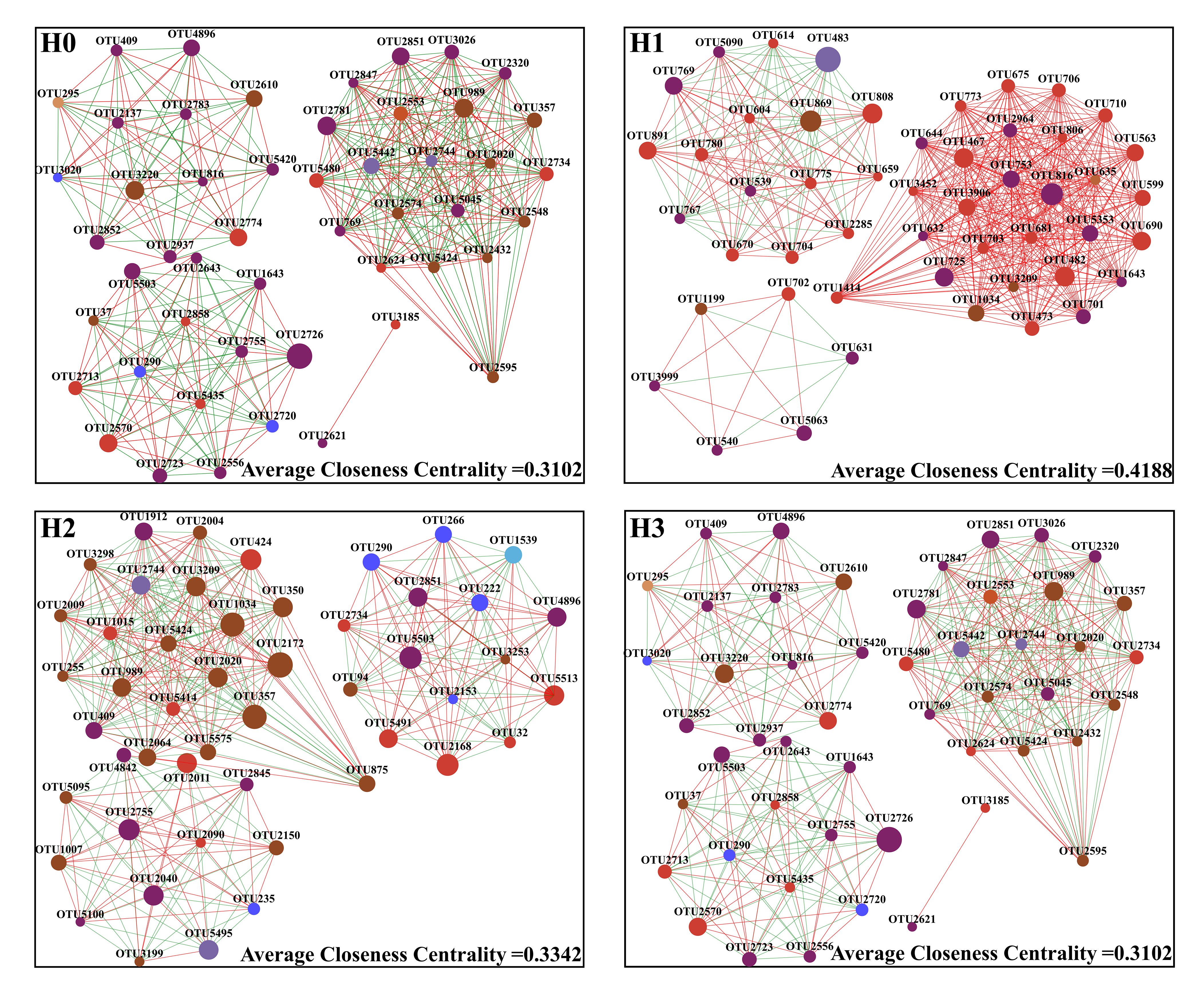


SI Fig. S3


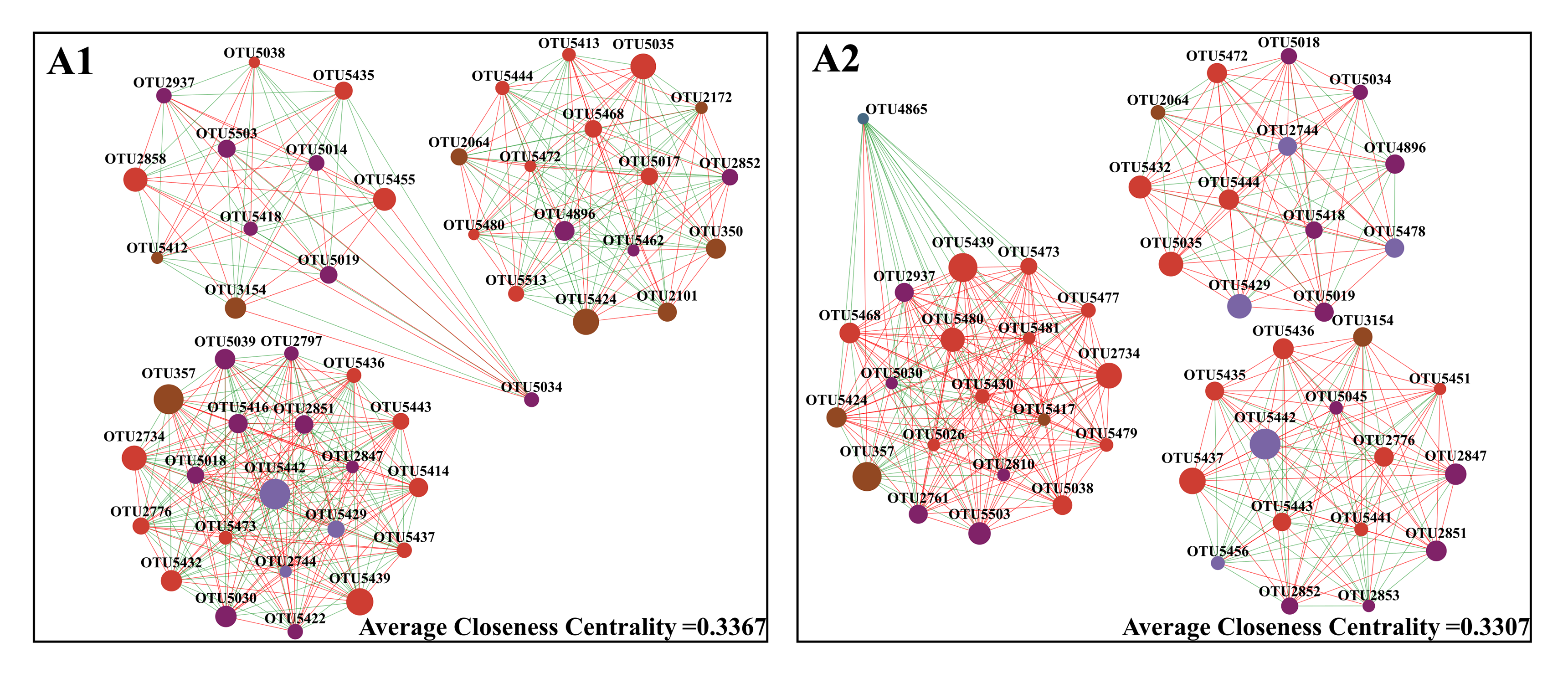


SI Fig. S4
